# A Case Report of Extracranial Intramuscular Cysticercosis As Soft Tissue Swelling in the Forearm and Thenar Eminence

**DOI:** 10.7759/cureus.94161

**Published:** 2025-10-08

**Authors:** Monika Singla, Arnav Galhotra, Neeraj Bansal, Raghav Chanday, Navreen Pooni

**Affiliations:** 1 Neurology, Dayanand Medical College and Hospital, Ludhiana, IND; 2 Medicine, Dayanand Medical College and Hospital, Ludhiana, IND; 3 Orthopaedics and Robotics Joint Replacement, Orison Hospital, Ludhiana, IND; 4 Radiology, Mayyo Imaging and Diagnostic Centre, Ludhiana, IND

**Keywords:** antiparasitic agents, extracranial cysticercosis, intramuscular, pseudotumor like, soft tissue swelling

## Abstract

Cysticercosis, caused by the larval form of *Taenia solium*, remains a significant public health issue in developing countries. Neurocysticercosis is one of the commonest clinical manifestations. Extracranial cysticercosis may present intramuscularly as a pseudotumor-like mass, making the diagnosis challenging. Isolated muscular cysticercosis, typically linked to measly pork, can also affect vegetarians, as was seen in our patients. Such presentations are uncommon and may mimic other soft tissue swellings. Its varied presentation marks it as an emerging concern globally. We present two cases, a seven-year-old girl and a 29-year-old male, of isolated cysticercosis, one in the thenar eminence and the other in the forearm, respectively, with no neurological symptoms. The lesions were responsive to antiparasitic therapy. Both patients could be managed medically; surgery was not required. This case highlights a rare and unusual manifestation of cysticercosis, expanding the spectrum of extracranial intramuscular involvement. It underscores the importance of considering soft tissue cysticercosis in the differential diagnosis, as well as the role of imaging in the unexplained swellings. This case also highlights the role of medical management in such cases.

## Introduction

Cysticercosis is a parasitic infection caused by the larval stage (cysticercus) of the pork tapeworm *Taenia solium*, endemic in resource-poor regions including India and increasingly recognized in high-income countries [1,2]. It also falls under the category of neglected tropical diseases [3]. Humans acquire it by ingesting tapeworm eggs through contaminated food or water. The larvae can migrate and form cysts in various tissues, including the brain (neurocysticercosis), muscles, skin, and eyes. Symptoms depend on the location and number of cysts and may include seizures, lumps, or vision problems. While the central nervous system remains the most affected site, extracranial cysticercosis, i.e., muscular and subcutaneous involvement, though less frequent, represents an important and often under-recognized clinical manifestation [2,4]. Among these, isolated soft tissue cysticercosis can present as painless or painful swellings that may mimic benign tumors, ganglion cysts, lipomas, or abscesses, leading to diagnostic confusion. Muscular cysticercosis typically manifests in three clinical forms: the myalgic form characterized by pain and tenderness; the mass-like or pseudotumor form presenting as a localized swelling; and the rare pseudohypertrophy type. In the upper limb, especially in regions such as the forearm and thenar eminence, such presentations are exceedingly rare and often misdiagnosed [5]. Given the functional importance of these areas, even small lesions can produce significant clinical symptoms.

High-resolution ultrasonography, MRI, and serological testing play crucial roles in early diagnosis [6]. A cyst with a well-defined wall and an eccentric echogenic nidus, representing the scolex, is considered pathognomonic. Treatment typically involves antiparasitic agents such as albendazole, sometimes combined with corticosteroids to reduce the inflammatory response. Surgical excision may be necessary in cases of diagnostic uncertainty, persistent symptoms, or when there is an inadequate response to medical therapy.

Herein, we report two rare cases of cysticercosis presenting isolated swellings in the forearm and involving the thenar eminence locations, which are rarely documented in the literature. Through these cases, we aim to highlight the importance of considering cysticercosis in the differential diagnosis of atypical soft tissue swellings in endemic regions.

## Case presentation

Herein, we report two rare consecutive cases of cysticercosis presenting as isolated swellings in the forearm and the thenar eminence, over a gap of a few weeks. These locations are rarely documented in the literature.

Case 1

**Figure 1:**
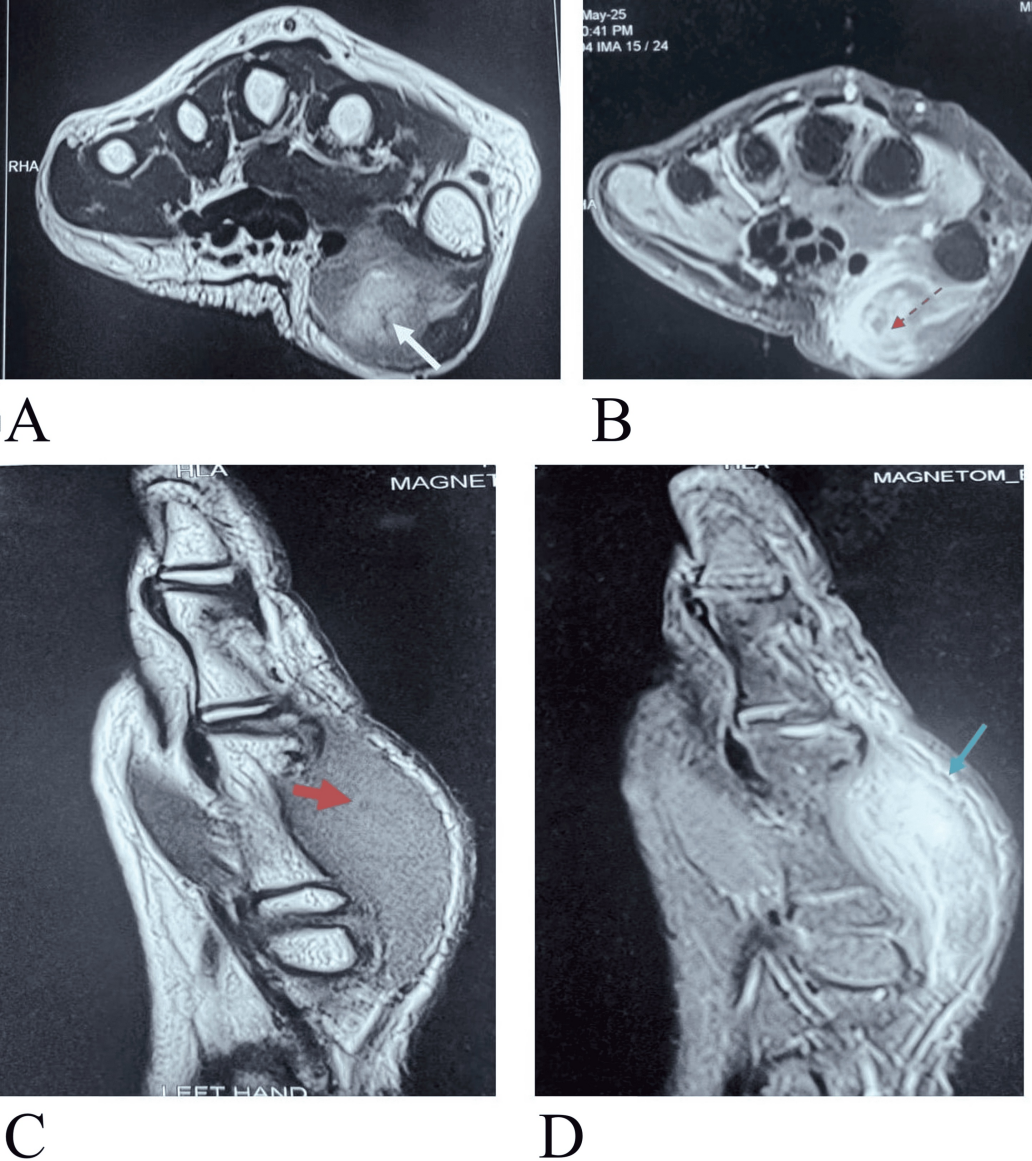
Left thenar eminence A) MRI of left hand, T2-weighted image (white arrow) shows heterogeneous cystic lesion in left thenar muscles with surrounding edema. B) T1-weighted post-contrast image (red dotted arrow) shows hyperintense lesion with marked surrounding edema. C) T1-weighted image shows hypointense lesion with hyperintense central dot suggestive of scolex (red thick arrow) with bulky left thenar muscles. D) MRI image shows bulky muscles (blue arrow) with intense surrounding edema.

A seven-year-old vegetarian girl was brought to the outpatient clinic of the Department of Neurology at Dayanand Medical College and Hospital, Ludhiana, Punjab, India, with the complaint of pain and swelling in her left thumb for the last five days in the first week of May 2025. The onset of symptoms was gradual, with the swelling and pain increasing progressively. The child reported difficulty in using the affected hand for routine activities due to discomfort. There was no history of trauma, fever, or recent infection. On physical examination, the swelling was noted at the base of the left thumb. It was 5 × 6 cm in size. It was tender to the touch, with mild restriction of movement of the joint. There was no redness or discharge. No similar swellings were present elsewhere on the body. The child had no history of similar complaints in the past, and there were no symptoms such as headache, visual disturbances, or seizures. She underwent an MRI of the left thumb (Figure 1). In view of a non-enhancing cyst with marked surrounding edema, the possibility of a degenerative cysticercus was kept. Before starting albendazole, an MRI of the brain and examination of the eyes were conducted to rule out the presence of any intracranial or intraocular cysts, and the findings were normal. She was given oral steroids, prednisolone 1 mg/kg/day for two weeks, along with albendazole 15 mg/kg/day for 15 days. She started responding very well to medical management. She was kept on regular follow-up biweekly. Her swelling and pain regressed significantly. Over 12-16 weeks, the swelling subsided completely. There was no pain or swelling thereafter.

Case 2

A 29-year-old male presented to the outpatient clinic of the Department of Orthopaedics with a complaint of swelling in the left forearm for the past 10 days in March 2025. The swelling was located on the forearm, approximately 3 cm distal to the elbow. The patient reported that the swelling appeared suddenly and gradually increased in size over the 10-day period. There was no history of trauma, insect bite, or infection at the site. He was a vegetarian. The patient was referred to the Department of Neurology for neurological evaluation and management. On clinical examination, the size of the swelling was 8 × 10 cm. It was soft and non-tender. There was no redness or signs of inflammation. The patient reported no pain or discomfort at rest or with movement. No other swellings were noted on the body. The patient had no history of systemic symptoms such as fever, headache, seizures, or visual disturbances.

**Figure 2:**
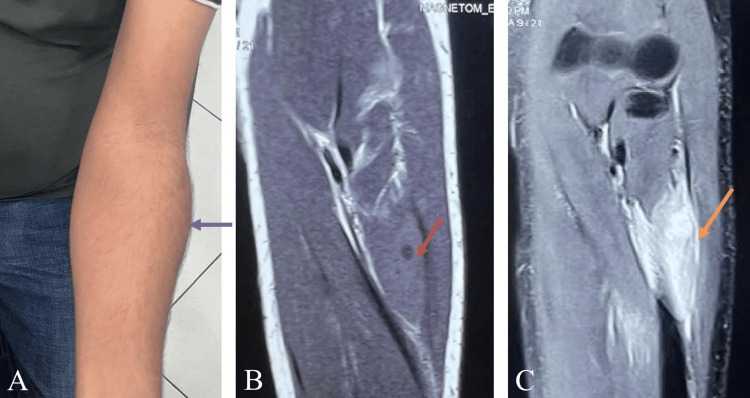
Left forearm A) Left forearm marked swelling near elbow (purple arrow). B) MRI T1-weighted image of left forearm shows cystic lesion with hypointense center with surrounding hyperintense area suggestive of scolex (red arrow). C) Intense contrast enhancement in left forearm muscles (orange arrow).

MRI of the left elbow (Figure 2) showed a non-enhancing cyst in the left supinator muscle with marked surrounding edema consistent with the possibility of degenerative cysticerci. MRI brain and eye examination was done to rule out any intracranial or intraocular cyst, which was found to be normal. He was started on oral steroids, prednisolone 60 mg/day for two weeks, along with albendazole 400 mg twice a day. With medical management, swelling and pain started regressing. Because of persistent pain, albendazole was continued for 21 days. He was kept on regular follow-up every two weeks for the same. The swelling subsided over six months. The patient is symptom-free after that.

**Figure 3:**
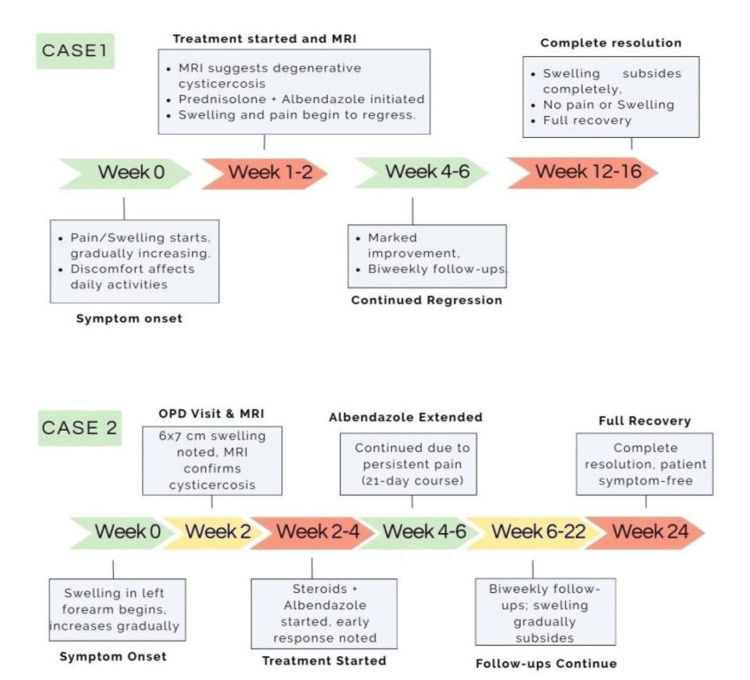
Timeline of the management of both cases, highlighting key events and milestones in the patients' journey from symptom onset to full recovery, including diagnosis and treatment interventions

Figure 3 illustrates the timeline of the management of both cases, highlighting key events and milestones in the patients' journey from symptom onset to full recovery, including diagnosis and treatment interventions.

## Discussion

Soft tissue cysticercosis, resulting from the larval stage of *Taenia solium*, is an uncommon manifestation. Isolated soft tissue cysticercosis is uncommon and has been sporadically reported in muscles such as those of mastication, the neck, tongue, trunk, internal oblique, forearm, and biceps brachii. Our case involved a rare presentation, a forearm swelling located in the muscle as well as in the thenar eminence. Such presentations can mimic various benign and malignant soft tissue tumors, posing diagnostic challenges, especially in regions where cysticercosis is endemic. It may typically include lipomas, sarcomas, myxomas, neurofibromas, or inflammatory lesions like myositis. Cysticercosis should be considered as a potential cause, as it is treatable and diagnosable using non-invasive methods like ultrasound and MRI. This case report describes a rare presentation of isolated soft tissue cysticercosis in the forearm of a 21-year-old male, who presented with a painless swelling initially mistaken for a soft tissue tumor or lipoma. Imaging (ultrasound and MRI) revealed a cystic lesion with a scolex, confirming the diagnosis of cysticercosis. The patient was successfully treated with anti-parasitic medication (albendazole) and steroids, leading to the resolution of the swelling. Clinicians should consider cysticercosis in the differential diagnosis of isolated soft tissue swellings, especially in endemic regions. Early and accurate diagnosis using non-invasive imaging can prevent unnecessary surgical interventions [5]. A patient presented with a forearm swelling initially suspected to be a soft tissue tumor. Imaging revealed a cystic lesion suggestive of intramuscular cysticercosis, and the diagnosis was confirmed via ultrasound and serological tests. The case highlights the importance of considering parasitic infections in soft tissue masses [6]. Another case discusses a rare presentation of subcutaneous cysticercosis in the scapular region of a patient who presented with a painless swelling. Initial clinical suspicion leaned toward a soft tissue tumor. Subcutaneous cysticercosis can mimic benign soft tissue tumors, even in uncommon locations like the scapula. A high index of suspicion and appropriate diagnostic tools are essential to avoid misdiagnosis and unnecessary interventions [7].

One case series reported multiple instances of isolated intramuscular cysticercosis, a rare and often misdiagnosed parasitic infection. Patients presented with localized, painless swellings in different muscles, initially suspected to be soft tissue tumors. Diagnosis was confirmed through imaging modalities like ultrasound and MRI. All patients responded well to conservative medical management with antiparasitic therapy, avoiding the need for surgery, like our patient [8]. Another case report described a patient with a painless swelling in the biceps muscle, initially suspected to be a soft tissue tumor. High-resolution ultrasonography revealed a well-defined cystic lesion with an echogenic scolex, leading to a definitive diagnosis of isolated intramuscular cysticercosis. The patient was successfully treated with anti-parasitic medication without surgical intervention, as our patients were managed conservatively. High-resolution ultrasound was found to be a valuable, non-invasive tool for accurately diagnosing intramuscular cysticercosis, allowing for prompt and effective medical management, while avoiding unnecessary procedures [9].

When evaluating soft tissue swellings in the muscles, like in the forearm, leg, thenar region, etc., several conditions should be considered in differential diagnosis [5,6] (Table 1). Table 2 shows the differential diagnosis of soft tissue swellings in muscles

**Table 1:** Various modalities to diagnose soft tissue swellings

Diagnostic Tool	Description
Ultrasonography (USG)	Non-invasive and cost-effective; identifies cystic lesions with a characteristic echogenic scolex, suggestive of cysticercosis.
Magnetic Resonance Imaging (MRI)	Provides detailed soft tissue contrast; reveals cystic lesions with specific signal characteristics. Useful for evaluating lesion relationships and identifying the scolex.
Serological Tests	Includes enzyme-linked immunosorbent assay (ELISA) and enzyme-linked immunoelectrotransfer blot (EITB); detects antibodies against Taenia solium, supporting the diagnosis.
Fine-Needle Aspiration Cytology (FNAC)	May show parasitic fragments or inflammatory cells. Sensitivity can vary, but it can aid in diagnosis.
Histopathological Examination	Gold standard for diagnosis; confirms presence of cysticercus larvae within the tissue.

**Table 2:** Differential diagnosis of soft tissue swellings in the muscles

Conditions	Clinical Features
Lipoma	Soft, mobile, painless mass; typically benign. Deep-seated lipomas may be mistaken for more serious pathologies.
Epidermoid (Inclusion) Cyst	Dome-shaped, subcutaneous mass with possible central punctum. May become inflamed or infected, leading to pain and tenderness.
Neurofibroma and Schwannoma	Benign nerve sheath tumors; firm, painless masses. Schwannomas are rare in the hand but can occur in the thenar region.
Soft Tissue Sarcoma	Malignant tumor; presents as a painless, enlarging mass. Rapid growth, firmness, and fixation to underlying structures are concerning features.
Pyomyositis and Abscess	Infectious; causes localized muscle inflammation and pus collection. Often associated with systemic signs of infection (e.g., fever).
Tuberculous Lymphadenitis	Chronic, painless lymph node swelling. Common in TB-endemic areas and may mimic soft tissue tumors.

High-resolution ultrasonography is a valuable tool, often revealing the characteristic cyst with an echogenic mural nodule (scolex). MRI complements this by identifying lesion location and staging, although the scolex is more distinctly visualized on ultrasound. Serological tests, such as enzyme-linked immunoelectrotransfer Blot (EITB), are highly specific, while ELISA may yield false negatives in solitary lesions. The various treatment options depend on the lesion's size, location, symptoms, and the presence of complications. Medical therapy in the form of anti-parasitic agents like albendazole (10-15 mg/kg/day for 15-30 days) or praziquantel is effective, especially for small, asymptomatic lesions [10]. Concomitant corticosteroids may be administered to mitigate inflammatory responses during treatment. Management of cysticercosis remains debated, especially in neurocysticercosis, where anticysticidal therapy may worsen inflammation and must be used cautiously. Preventive measures, including improved hygiene, proper food handling, and treating intestinal infections, are essential to control the spread. Surgical excision is indicated for lesions causing significant symptoms, those not responding to medical therapy, or when the diagnosis is uncertain. Complete excision not only provides symptomatic relief but also allows for definitive histopathological diagnosis. 

In our cases, the decision between medical and surgical management was individualized based on the lesion's characteristics and the patient's clinical presentation. Both patients responded quite well to conservative management.

The limitation of this case was that the diagnosis of cysticercosis in both cases was presumptive. The biopsy could not be performed as the patients were not willing.

## Conclusions

Extracranial cysticercosis as soft tissue swellings, though rare, should be considered in the differential diagnosis of isolated soft tissue swellings in endemic regions. This case highlights the rare presentation of intramuscular cysticercosis in the forearm and the thenar eminence, the unusual sites where it clinically and radiologically mimicked a soft tissue tumor. Such atypical localization without systemic involvement or neurological symptoms can lead to misdiagnosis and unnecessary interventions. The case underscores the importance of considering parasitic infections like cysticercosis in the differential diagnosis of soft tissue swellings, especially in endemic regions. This case contributes to the limited literature on extracranial intramuscular cysticercosis, providing valuable insights into the diagnosis and management of such a rare presentation. Accurate diagnosis through imaging, especially high-resolution ultrasound or MRI, can prevent misdiagnosis and avoid unwarranted surgical procedures. Early identification and appropriate antiparasitic treatment can lead to complete resolution and favorable outcomes.
